# Migraine self-management at work: a qualitative study

**DOI:** 10.1186/s12995-024-00421-w

**Published:** 2024-06-04

**Authors:** Clara Knauf, Katherina Heinrichs, Rolf Süllwold, Andrea Icks, Adrian Loerbroks

**Affiliations:** 1 Institute of Occupational, Social and Environmental Medicine, Centre for Health and Society, Faculty of Medicine, University of Düsseldorf, Moorenstr. 5, Düsseldorf, 40225 Germany; 2 Institute of Health and Nursing Science, Charité – Universitätsmedizin Berlin, Corporate Member of Freie Universität Berlin and Humboldt-Universität zu Berlin, Berlin, Germany; 3 Berolina Klinik, Löhne, Germany; 4 Institute of Health Services Research and Health Economics, Centre for Health and Society, Faculty of Medicine, University of Düsseldorf, Düsseldorf, Germany

**Keywords:** Migraine, Qualitative research, Self-management, Workplace, Headache, COVID-19 pandemic

## Abstract

**Background:**

Migraine is common and can be highly disabling. Adequate migraine self-management (SM) can mitigate the potentially adverse health effects of migraine. However, adequate SM can be challenging to implement in everyday life, for instance, at the workplace. We aimed to explore how migraine SM is carried out at work and which occupational factors may determine effective implementation according to employees with migraine. We also explored the potential impact of the COVID-19 pandemic and the associated re-arrangement of working conditions on migraine SM at work.

**Methods:**

We conducted semi-structured qualitative interviews (08/2020–01/2021). Participants were eligible if they have worked for at least six months with a minimum of twenty hours per week and with a migraine diagnosis. The interviews were transcribed and content-analyzed using MAXQDA.

**Results:**

Migraine SM was perceived to be influenced by social interactions at work (e.g., in terms of understanding vs. stigmatization), the level of job decision latitude (JDL, i.e., the extent to which one is able to influence work processes, e.g., when working from home), and workplace design (e.g., in terms of opportunities to withdraw from work). During the COVID-19 pandemic, especially increased JDL appeared to favorably influence migraine SM.

**Conclusions:**

Migraine SM at work is fostered or complicated by various psychosocial working conditions. By considering these facilitators and barriers, more migraine-friendly workplaces can be created.

**Supplementary Information:**

The online version contains supplementary material available at 10.1186/s12995-024-00421-w.

## Background

Migraine is associated with a considerable burden in terms of its prevalence, related disability, and cost. The prevalence is especially high among people between the age of 36 and 46, who are in the midst of their occupational life [1]. It has been estimated that the European Union loses € 111 billion annually due to migraine [2]. As much as 93% of this amount is considered to be attributable to indirect cost, i.e., cost resulting from lost and reduced productivity at work [2].

To successfully manage their condition, individuals with migraine need to acquire and apply various skills (i.e., self-management [SM]). Five major areas of migraine self-management activities have been identified in prior studies: (1) utilizing the healthcare system, (2) taking medication adequately, (3) using alternative therapies (such as osteopathy, herbal, and homeopathic remedies), (4) requesting social support, and (5) self-care (e.g., trigger detection and avoidance, stress management, and a healthy lifestyle) [3–5].

Migraine SM can be hampered or facilitated by external factors in everyday life, for instance, at the workplace. With regard to the latter, the European Federation of Neurological Associations (EFNA) conducted a survey in 2020 inquiring individuals with migraine or other types of headaches (*n* = 167) from 20 European countries what their company should do to help them cope better with the effects of their health condition [6]. Proposed areas of improvement included (but were not limited to): more understanding by managers, more opportunities to work from home, private workspace, less social interaction, part-time work, and noise-cancelling headphones [6]. In the same vein, Scaratti et al. [7] used an online questionnaire to examine the needs of headache patients in Europe (*n* = 103) related to staying at or returning to work. Here, too, physical environment adaptions (such as single offices, rest rooms), work-related aspects (e.g., longer, and flexible breaks), and support at work (among other things, social support by supervisors and human resources) were mentioned [7].

While there is thus confirmatory preliminary evidence regarding headache SM, there is still a need to examine in depth if and how working conditions may affect successful implementation of specifically migraine SM (rather than headache management in general) at work. Migraine cannot be equated with headache, as headache is only one symptom of migraine, which is also usually accompanied by other symptoms such as nausea, vomiting, or sensitivity to light, and thus likely to lead to more impairment. We aimed to gain detailed insights into the types of working conditions that may facilitate or hamper the ability to self-manage migraine. Work arrangements have changed swiftly and dramatically for many employees during the COVID-19 pandemic and we therefore also sought to explore the potential effects of these re-arrangements on SM strategies.

## Methods

We conducted a qualitative study using semi-structured interviews. Our report adheres to the Consolidated Criteria for Reporting Qualitative Research (COREQ) statement [8].

The primary researcher (CK) was a medical student at the time of our study. Qualitative research skills are usually not acquired during medical studies in Germany. CK conducted the study to obtain a German Doctor of Medicine (Dr. med.) degree, which is comparable (in terms of its scope and quality) with a master thesis rather than with a PhD degree. The general choice of self-management at work as the research topic was due to the foci of the research group headed by AL, who acted as CK’s thesis supervisor. It was CK’s wish to relate her research project to migraine, as she had several migraine patients in her personal environment. CK familiarized herself extensively with the methodology of qualitative research (especially through self-studies and online tutorials) and in particular with the coding process. Prior to the interviews and the analysis, CK was trained (e.g., in interviewing techniques) by KH who is an experienced qualitative researcher [9, 10] (female, degrees in psychology and public health). Also, detailed feedback and opportunities for reflection were provided during the actual interviewing and subsequent analysis (see below).

### Study population

We sought to recruit participants who met the following inclusion criteria: (1) participant-report that migraine has ever been diagnosed by a physician and (2) employment with the migraine diagnosis for at least six months with a minimum of 20 working hours per week. Study participants were recruited via three different pathways: an inpatient rehabilitation clinic for headache disorders (Berolina Klinik, Löhne, Germany), migraine self-help groups from different regions in Germany, and private contacts of members of the study team. In the run-up to the interviews all potential participants received information on the objectives of the study, the professional background of the interviewer (CK) and the inclusion criteria (either via a flyer or from CK in person). This information was presented again later at the start of the interviews. It was also ensured at the beginning of the interviews that the inclusion criteria were met. All participants provided written informed consent prior to the interviews. In line with participants’ preferences, interviews were either conducted face-to-face or by telephone. Face-to-face interviews took place in the clinic or at the participants’ homes. We did not gather any information from non-participants, that is, from those who were exposed to our recruitment efforts (e.g., members of the self-help groups), but decided not to participate. Our study was approved by the Institutional Review Board of the Faculty of Medicine of the University of Düsseldorf, Germany (# 2019 − 627).

### Data collection

Based on previous qualitative research by our group [9, 11], we designed a topic guide [see Additional File 1]. After three interviews, the topic guide was discussed and adjusted by the study team. In particular, we then decided to add one question about migraine SM at work specifically during the COVID-19 pandemic, as this was an issue frequently referred to in the first interviews. Prior to the interviews, participants were asked to complete a standardized questionnaire collecting information on socio-demographics and the health status (see Results – Table 1).

Data collection took place from August 2020 to January 2021. All interviews were conducted in German by one researcher (CK), who took field notes during the interviews. Besides the interviewer and the participant, no one was present. Follow-up interviews were not conducted. All interviews were digitally recorded and transcribed. CK received detailed feedback on the way she conducted the interviews after three interviews from AL, who is an experienced qualitative researcher [9–11, 13] (male, degrees in epidemiology and health sciences). These three interviews were initially supposed to serve as test interviews, but as they contained valuable information we decided to include these interviews in the analysis. There were also no major concerns from AL regarding CK’s interview style except that CK should be even more careful not to ask too closed questions. Data collection was terminated when thematic saturation was reached, which implies that no new information was expected to be delivered by additional interviews [14]. To verify that saturation had been reached, the data analysis already started during data collection. As the interviews and the analysis were carried out by the same person (CK), she was able to pay close attention to when no new aspects were mentioned in the interviews. The interviews were then terminated after consultation with AL. Study participants were not given access to the transcripts, nor could they provide feedback on the findings.

### Qualitative data analysis

Transcripts were content-analyzed [15] using the software MAXQDA 2020. The coding was based closely on the topic guide and the research questions of the study. Consequently, the questions of our topic guide [see Additional File 1] served as main categories (deductive coding). For example, the question ‘Are there conditions at work that help you deal with your migraine?’ served as one main category, labelled ‘Facilitators of migraine self-management’. Subcategories were then developed based on the interview content (inductive coding). After CK had coded five interviews, the coding framework was carefully reviewed by two experienced qualitative researchers (KH and AL). Once everyone approved the initial coding framework, CK applied the code system to all interviews and expanded it by adding further categories. After this first round of coding was completed, AL reviewed the codes again. For instance, AL checked the structure of the coding tree level by level to see whether the codes may be overlapping or seemed ordered logically (e.g., according to the same criteria) within each level. He read all text passages included into each code to explore whether the respective text passages relate to the same phenomenon (and thus can be grouped into a single code) or whether codes may be merged or could be further sub-divided. Based on this, he suggested changes and other inductive categories and discussed them with CK. Based on this discussion, CK re-coded all interviews. Afterwards, the codes were re-discussed with AL and the framework was marginally adjusted. Finally, CK carried out a third and final round of coding. The analysis was carried out using German-language transcripts. The quotes presented in this paper were translated from German into English by a researcher who is familiar with health research and has a Master’s degree in English studies (see acknowledgments).

## Results

### Description of the sample

In total, 24 interviews were conducted with a mean duration of 31.8 min (range: 17.6–55.3, standard deviation [SD] = 11.4). Twelve interviews were carried out face-to-face and twelve by telephone. Ten participants were recruited in the rehabilitation clinic, nine through self-help groups, and five were private contacts of study team members. Table 1 shows characteristics of our sample: our study population was mainly female (88%), and the mean age was 49.5 years (SD = 9.0). More than half of the participants (*n* = 14) were classified as having a job that was mainly characterized by cognitive or psychosocial demands (e.g., librarian, social worker, pastoral counsellor). The remainder of participants had jobs with mixed requirements (e.g., teacher, nurse, shop assistant). The mean time since the diagnosis of migraine was 20.6 years and varied from five to 40 years (SD = 8.5). On average, participants reported to have had 10.0 days of migraine during the last month, but the variation was considerable (range: 2–26, SD = 6.6).

**Table 1:** Characteristics of the study population (*n* = 24)

Domain	Characteristics	
Socio-demographics	Female sex, n (%)	21 (87.5)
	Age in years, mean (standard deviation), min - max	49.5 (9.0), 31–62
	High educational level^a^, n (%)	14 (48.3)
Occupational data	Current/last job demands mainly cognitive/psychosocial^b^, n (%)	14 (58.3)
	Working full-time (vs. part-time/no current job), n (%)	10 (41.7)
	Work stress^c^ (0–10), mean (standard deviation), min - max	6.8 (2.0), 3.5–10
	Limitation of work due to migraine^c^ (0–10), mean (standard deviation), min - max	6.6 (2.9), 1–10
	Limitation of migraine management due to work^c^ (0–10), mean (standard deviation), min - max	6.4 (3.1), 0–10
Migraine-related data	Migraine without aura (vs. migraine with aura or mixed form), n (%)	12 (50)
	Years since migraine diagnosis, mean (standard deviation), min - max	20.6 (8.5), 5–40
	Migraine days during the last month, mean (standard deviation), min - max	10.0 (6.6), 2–26
Further health data	Relaxation procedures are carried out regularly, n (%)	12 (50.0)
	Obesity (BMI^d^ ≥ 30 kg/m²), n (%)	6 (25.0)
	Other chronic disease(s)^e^, n (%)	15 (62.5)
	Depression (PHQ-2^f^ ≥ 3), n (%)	2 (8.3)
	Anxiety (GAD-2^g^ ≥ 3), n (%)	9 (37.5)

^a^’Abitur’ or ‘Fachhochschulreife’ (school degrees that make graduates eligible for higher education institutions) versus lower degrees or no formal degree.

^b^Job demands mainly cognitive/psychosocial ((e.g., librarian, social worker, pastoral counselor) as opposed to mixed job demands (cognitive/psychosocial and physical demands, e.g., teacher, nurse, shop assistant); variable constructed based on interview content.

^c^Numeric scale from 0 (low stress/no limitation) to 10 (high stress/high limitation).

^d^Body Mass Index. Calculated by dividing the body weight (in kilograms) by the body height (in meters) squared.

^e^Most frequent chronic conditions: anxiety or panic disorder (*n* = 14), depression (*n* = 6), arterial hypertension (*n* = 6), tension type headache (*n* = 4).

^f^Patient Health Questionnaire-2. Exceeding the cut-off value of 3 indicates depressive symptoms [12].

^g^Generalized Anxiety Disorder Scale 2. Exceeding the cut off value of 3 indicates anxiety [12].

### Qualitative interviews

A broad range of psychosocial facilitators and barriers of migraine SM at work emerged from our data which are described in the following. Our results indicated that the COVID-19 pandemic affected workplace SM both favorably and adversely.

All quotes referenced below can be found in the appendix [see Additional File 2]. If there is an interest in the shared migraine self-management strategies at work, a description of these can also be found in the appendix [see Additional File 3]. These are not explained in more detail below however, as the specification of the strategies is beyond the scope of our main research questions.

#### Which psychosocial working conditions influence migraine self-management at work?

**Facilitators**: The following working conditions were perceived to be helpful in managing migraine at work: (1) high social support, (2) high job decision latitude (JDL; i.e., the degree of an employee’s control over tasks and how and when they are addressed), and (3) a suitable workplace design.

Receiving social support at work from colleagues, supervisors, or in form of company or government policies was reported. Support by colleagues included the understanding for the illness, especially from colleagues with the same disease. It was frequently expressed that some colleagues could – without words – sense when the individual with migraine was not feeling well. Relevant support-related activities by colleagues that made migraine SM easier included support in avoiding triggers (e.g., by ensuring a good air supply), encouragement to withdraw during acute migraine attacks, and taking over tasks (quote 1). According to the participants, social support by supervisors was effective by creating flexible arrangements regarding tasks, working times, and locations, for example the option to work from home (quote 2). Overall, it seemed that understanding for the disease and the social support from colleagues and supervisors facilitated the SM strategy communication. In Germany, people with chronic illness (including migraine) can apply for a so-called “degree of disability”. This entails entitlement to – amongst other things – more holidays and better protection against dismissal from the job. This legal possibility was perceived as helpful, also in the way that these official degrees simplified the justification of the disease and certified its seriousness (quote 3). Furthermore, one participant shared that she has approached the staff council (in her case the teachers’ council). By disclosing her migraine in front of the council, she gained the understanding of her colleagues (quote 4).

In addition to social support, the study participants described a high JDL – that is, a high degree of control over their tasks – as beneficial. Influence on the order of tasks was perceived as allowing for flexibility in planning and carrying out SM during migraine attacks. Control over the type and number of tasks (e.g., working independently instead of attending meetings [quote 5] or the possibility to avoid screen work during the acute attack [quote 6]) were relevant in managing migraine attacks. Another important factor in terms of JDL was the possibility of working from home. The latter provided the opportunity to organize the working day according to one’s own preferences and to take flexible breaks. For example, one participant reported that one had the option of starting one’s working day later at home if one had a headache in the morning (quote 7). The improved opportunities of stopping work at home was also considered to be beneficial because, according to one participant, the threshold to stop working when experiencing complaints is lower when one works at home than at the workplace (quote 8).

A suitable workplace design, which referred mainly to a single rather than a multi-person office, was also experienced as helpful. In a single office, one had the opportunity to retreat and control air supply and light. As many individuals with migraine are sensitive to light during an attack, this can be beneficial (quote 9). In addition to the office situation, the provision of appropriate work equipment such as flicker-free screens, noise-cancelling headphones, and height-adjustable desks was also reported to have a positive effect on one’s migraine. The latter had been reported to reduce cramping in the shoulders and neck and thereby easing headaches (quote 10).

**Barriers**: The reported barriers represented in certain respects the opposites of the above-mentioned facilitators. Yet as these were explored separately and several aspects were not overlapping, they are described independently. The following aspects were mentioned: (1) poor social interactions, (2) unfavorable working time arrangements, (3) unfavorable workplace arrangements, and (4) other working conditions.

Poor social interactions included interactions with colleagues, supervisors, and service users. Several participants felt that migraine as a disease was often not taken seriously by others and stigmatized at their workplace. For example, this led to migraine being dismissed as a trifle or lack of understanding for staying at home in case of complaints (quote 11). One participant also emphasized the lack of empathy at the workplace: if one was present at work, one was expected to be fully functioning (quote 12). In this context, participants also found it bothering that migraine is “an invisible condition” (quote 13). During contact with service users (e.g., customers, patients, clients), it was reported to be disturbing that there were often high expectations that could not be met during a migraine attack and the associated impairments (quote 14).

Some workplace and working time arrangements were also considered as detrimental. In workplaces where migraine triggers were present (such as heavy noise, little air supply, bright light), migraine SM and especially the preventive strategy of trigger avoidance was reported to be negatively affected. For example, one participant reported the problem of sharing an office and not being able to adapt it to one’s own need, for example not having control over room temperature (quote 15). Visual display unit (VDU) work was also described to be a migraine trigger (quote 16). Another mentioned problem in workplace design was the lack of opportunities to retreat – physically (e.g., due to lack of break rooms, open-plan offices) and mentally (in terms of being permanently approachable). One participant, for example, shared that it was very difficult to deal with migraine if one always had to be approachable on business trips and thus has no possibility to retreat (quote 17). The lack of opportunities to retreat from challenging situations was believed to worsen symptoms and delay recovery from an attack. In terms of unfavorable working time arrangements, irregularity was mentioned as it implies an interruption of one’s usual circadian rhythm, which may trigger a migraine attack. This could be unscheduled client appointments due to public traffic (quote 18), shift work or exceptional weekend work (quote 19), but also business trips (including school trips as a teacher), missing or insufficient breaks, and time pressure at work (quote 20).

Other working conditions that were considered as barriers included, for example, a lack of staff and therefore a lack of replacement hampering one to go home when experiencing an acute migraine attack (quote 21) as well as poor contract conditions. Regarding the latter, one participant shared that she did not call in sick despite symptoms because then she did not get paid (quote 22).

It should also be mentioned that one study participant did not see any connection between migraine and the workplace and thus could not name any facilitators or barriers to SM (quote 23).

#### How was migraine self-management at work affected by the COVID-19 pandemic?

In Germany, the first two COVID-19-related lockdowns began in March 2020 (until May 2020) and in December 2020. As we gathered our data between August 2020 and January 2021, experiences during the COVID-19 pandemic were an important topic in the interviews.

One positive aspect for migraine SM during the pandemic was reported to be increased JDL. This was mainly due to new opportunities (and in some cases the obligation) to work remotely (quote 24) providing the advantage of more flexibility (e.g., the arrangement of breaks), a lower noise level, and an elimination of travel times. The fact that many employees took the opportunity to work from home also meant that the office was less busy and therefore more quiet (quote 25). This increased quietness also seemed to be beneficial for employees with migraine. One study participant reported that it was easier to close the office door to do a few stretching exercises (quote 26). The increased structuring of the working day and thus increased regularity as a facilitator for migraine SM (e.g., through stricter appointment policy [quote 27]), and the partial reduction of the workload also appeared to be positive. Furthermore, one study participant had more of a feeling of being needed in one’s work at a nursing home. The increased job satisfaction was reported to lower the frequency of migraine complaints (quote 28).

The stress caused by the additional hygienic measures, the mouth-to-nose covering, and the increased amount of screen work were perceived as negative for migraine SM. Coming in contact with COVID-19-positive people often necessitated use of additional stressful measures such as the application of hygiene or personal protective measures. This was, for example, reported by a study participant that worked as a nurse in a hospital (quote 29). The mouth-to-nose covering seemed to make it difficult not only to breathe but also to speak, which in turn was perceived to trigger migraine (quote 30). A final migraine trigger in the pandemic was the fear of the end of the pandemic and thus the loss of the possibility to work from home (quote 31).

## Discussion

### Summary of main findings

According to our participants migraine SM at work is affected by social interactions (e.g., understanding as a facilitator vs. stigmatization as a barrier), the extent of JDL (e.g., in terms of working hours and localization) as well as the workplace design (e.g., regarding opportunities to retreat or to avoid VDU work). During the COVID-19 pandemic, it was considered positive for migraine SM that the daily structure was associated with more predictability and planning (e.g., through stricter appointment scheduling). It was also emphasized that there were more opportunities to work from home and thus better conditions for appropriate migraine SM through more JDL. A negative aspect associated with the COVID-19 pandemic was increased work-related screen time. Participants also shared that the novel hygiene measures (e.g., after contact with a COVID-19-positive person) and the mouth-to-nose covering triggered migraine complaints.

### Findings in light of earlier research

In terms of barriers and facilitators, our findings are in line with prior research. A qualitative study on migraine and chronic daily headache management by Peters et al. [4] highlighted the importance of social support. The authors concluded that social support, especially from peers with the same conditions, can lead to better understanding from colleagues. The lack of social support and the feeling of stigmatization was an important aspect in another qualitative study by Heidari et al. [16], focusing on common themes of migraine patients. In that study one participant reported going to work despite migraine, because the supervisor seemed not to take migraine seriously [16]. One study – in accordance with our findings – linked stigmatization to migraine being an invisible disease, limiting the understanding for the condition [17]. Other factors influencing migraine SM that emerged from our study were factors related to workplace design. This is in keeping with findings from a cross-sectional study on the burden and impact of migraine on work productivity and quality of life that also addressed job-related migraine triggers and coping strategies: Looking at computer screens for too long was one of the two most frequently mentioned migraine triggers at the workplace [18]. Having control of light, noise and smells was under the top five coping strategies [18].

In the context of the EFNA study [6], individuals with migraine and other headache type patients were asked what their company should do to help them cope better with their condition. The wishes mentioned included a greater understanding for the disease, less social interaction, the possibility to work from home, flexible working hours, and more opportunities to withdraw if needed [6]. These aspects overlap with the facilitators that emerged from our study, in particular regarding social support and JDL.

Regarding the COVID-19 pandemic, our results are consistent with findings from other studies that found a link between personal protective equipment, especially the wearing of masks, and a worsening of migraine [19–21]. There is also further evidence that the increased screen time, for example due to remote working or online lessons during the pandemic, served as a trigger for migraine, in particular in young adults and adolescents [22, 23]. However, remote working during the pandemic was generally considered to have a positive impact on migraine (e.g., reduced migraine attack duration) [24]. In a qualitative study by Buse et al. [20], examining the general impact of the COVID-19 pandemic on patients with migraine, participants reported that working from home was associated with more control, e.g., over the work environment. This reflects the importance of JDL for migraine SM. Notably, some factors that were mentioned to influence SM in general (e.g., social support) played little or no role in the COVID-19 pandemic and associated SM. The pandemic served as a kind of natural experiment elucidating which factors – when modified – influence migraine and its SM. Based on this, it can be hypothesized that the facilitator “JDL,” which played an important role in general and during the pandemic, has the utmost relevance on migraine SM at work. To our knowledge, no previous qualitative study on migraine or chronic headache has yet highlighted the high relevance of JDL for SM.

### Methodological considerations

The interviews were conducted face-to-face or by telephone, depending on the preference of the participants. This provided us with the opportunity to include participants nationwide and despite restrictions due to COVID-19. We did not notice considerable differences regarding the contents between the two interview modes, which is supported by earlier research [25]. To reduce a potential healthy worker bias (i.e., the assumption that the working population is healthier than the non-working population), we recruited migraine patients who had ever worked for six months with a diagnosis of migraine and not only patients who were currently working. This allowed us to include patients who might have had to leave their job due to severe migraine or who attempted to regain their workability through rehabilitation.

We relied on the patients’ report of being diagnosed with migraine by a physician, and we did not apply the International Classification of Headache Disease [26] for diagnoses. However, those participants who were recruited from the rehabilitation clinic for migraine (*n* = 10) had received a medical diagnosis of migraine and their condition was severe enough to threaten their employment status. Further, as we interviewed mainly patients from this rehabilitation clinic and from self-help groups, however, we cannot exclude the possibility of a selection bias: those patients interact with other individuals with migraine, a are usually well-informed about their condition, and their experiences and perspectives may differ to some extent from the broader patient population. Moreover, only three out of 24 participants were male. This may have limited the scope of views that emerged from male participants regarding migraine SM. Also, our study especially included patients who worked in a job with mainly cognitive and social demands. It is well conceivable that their experiences differ from that of migraine patients who work in a job with mainly physical demands (e.g., individuals working in transportation or farming). We were able to cover a broad distribution regarding the average number of migraine days per month (mean = 10.0, SD = 6.6, range: 2–26 days). These observations increase the confidence that we covered a large range of potential views and experiences.

Another methodological weakness is that the coding was carried out by only one single person who had no previous experience in coding (CK). The intense involvement of additional individuals in the coding process (e.g., more experienced coders, people with migraine, occupational physicians, and/or neurologists), would likely have led to a richer analysis and additional insights, but this was beyond the resources of our study (i.e., time and financial means).

Finally, due to the limited experience with qualitative research methods of the first author, who was also the primary analyst, and due to the fact that it was not feasible to substantially involve additional analysists in the coding, the depth of our analyses may have been limited. Accordingly, our study may be classified as a ‘topical survey’ with aspects of a ‘thematic survey’ – according to the classification of findings in qualitative studies suggested by Sandelowski & Barroso [27]. A topical survey stays close to the data collected and is primarily a description of it, whereas a thematic survey provides a higher level of transformation of data [27]. The purpose of thematic surveys were only achieved to a limited extent. However, Sandelowski & Barroso state that a topical survey is not necessarily inferior in terms of the quality of its value [27].

### Implications for practice and research

Based on our findings, interventions could be devised to improve migraine SM at work. Regarding social support, it is important to reduce stigma of migraine to create a working environment in which patients feel comfortable to talk openly about their migraine without it being dismissed as a trifle. Our study thus calls attention to the fact that migraine healthcare professionals should offer support for improving patients’ social communication skills in the workplace leading to greater acceptance of the condition. One health care sector that seems particularly suitable for this endeavor is rehabilitation. Treatment in rehabilitation clinics in Germany involves patient education, which can help to raise awareness among migraine patients for the potential influence of their working conditions on their opportunities to manage their migraine at work. Also, patients can be empowered (e.g., by improving knowledge about legal frameworks and practicing communication skills) to modify their working conditions to some extent. Similar concepts are currently tested for other conditions than migraine [28].

To increase JDL, employers should try to give migraine patients as much freedom as possible. For example, for office jobs the possibility of expanding remote working should be explored. Here, the experience gained during the COVID-19 pandemic can be used. If working from home is not possible, care can be taken to create a migraine-friendly workplace, for example by providing single offices, noise-cancelling headphones, height-adjustable desks, and places of retreat. VDU work could also be designed to be as gentle as possible, e.g., by using flicker-free screens. If available and needed, occupational physicians should support all these interventions by educating workers with migraine and by serving as mediators between supervisors and employees with migraine.

All these interventions should be carefully developed and evaluated prior to their implementation in routine care. We believe that more preparatory research is needed. Firstly, as mentioned above, our analysis may be limited in depth. It therefore seems promising to carry out additional qualitative studies that involve analysts with more diverse professional backgrounds and employees with migraine as co-researchers. In the next step, the scope of the problem could be confirmed, and possible interventions may be explored. Quantitative research (e.g., surveys) would be suitable to examine, amongst others, the proportion of workers with migraine that find self-management at work to be challenging, to prioritize areas for intervention, and to examine what types of interventions would be acceptable to those receiving them and those potentially delivering them. Also, working life in the post-COVID-19 era has further evolved since our study to arrive at a “new normal” (e.g., allowing for more home office working hours than in the pre-COVID-era), which our study does not reflect, and which follow-up qualitative studies could explore. Furthermore, quantitative studies could test hypotheses that can be deduced from our qualitative study (e.g., “The ability to perform migraine SM at work is associated with the level of experienced JDL”). Such research could move beyond self-management as an outcome to include symptoms and occupational outcomes (e.g. workability, presenteeism and absenteeism) and may explore whether improved migraine SM at work curtails the considerable cost associated with migraine-related impairment.

## Conclusions

Migraine SM at work is influenced positively and negatively by various occupational factors. By considering these facilitators and barriers, a more migraine-friendly workplace can be created to reduce a burden not only for patients but also for society. Further research is needed before interventions can be implemented.

### Electronic supplementary material

Below is the link to the electronic supplementary material.

Supplementary Material 1

Supplementary Material 2

Supplementary Material 3

## Data Availability

Data cannot be shared publicly because the transcripts may contain sensitive information. The data may be obtained from the corresponding author upon reasonable request and provided that legal frameworks are not violated and that responsibilities and confidentially have been clarified.

## Abbreviations

CM: Chronic migraine
SM: Self-management
JDL: Job decision latitude
EFNA: European Federation of Neurological Associations
VDU: Visual display unit
