# Supplementary material for: Migraine self-management at work: a qualitative study
Source: J Occup Med Toxicol. 2024 Jun 4;19:22. doi: 10.1186/s12995-024-00421-w (PMC11149347; doi:10.1186/s12995-024-00421-w)
Supplement: Supplementary file 1 — Supplementary Material 1 [file 12995_2024_421_MOESM1_ESM.docx]

**Additional File 1 - Topic guide**

| **Initial questions about the workplace** | You told me you work as _[xxx]_ and have had migraine for _[xxx]_ years.   - How would you describe a typical day at work? - Has the COVID-19 pandemic changed your work routine in any way? If yes, how? |
| --- | --- |
| **General questions about the disease** | - To what extent does your migraine trouble you in everyday life? - How do you know that a migraine attack is approaching? (e.g., visual disturbances, sensory disturbances, speech disturbances) - How soon before the beginning of the migraine attack do these symptoms occur? How long do they last? - Is your migraine attack linked to certain triggers? (e.g., climatic conditions, stimulants, changes in sleep-wake rhythm, stress ...) - What symptoms do you suffer from during a fully developed migraine attack? |
| **Self-management strategies in everyday life** | We have already talked a bit about your migraine symptoms and your treatment situation (short questionnaire). Now let’s talk about what you do to handle your migraine, for example, to prevent attacks or respond appropriately to them. This could include doctor’s visits, taking medications on time or regularly, alternative therapies, avoiding triggers, stress management, an overall healthy lifestyle, and many other practices that help you manage your migraine.   - What do you do in everyday life, i.e., independently of work, so that your migraine does not bother you too much?   - What measures do you take when a migraine attack is approaching?   - What do you do if these measures fail to avert an attack, i.e., if you have an acute migraine attack?   - With whom do you talk to about your migraine? Who supports you in dealing with your migraine? |
| **Implementation of self-management at work** | - To what extent can you implement these measures in your work routine? |
| **Facilitators** | - Are there conditions at work that help you deal with your migraine? If so, what are they?   - How much freedom do you have to design work processes yourself and adjust them to your health needs? Examples?   - Have you disclosed your migraine at work? Why did (or didn’t) you disclose your condition?   - Are there people at work who support you, and how does this support look like? |
| **Barriers** | - What conditions at work make it difficult for you to deal with your migraine?   - What do the specific situations look like in detail?   - What actions are particularly difficult to implement then? Why?   - What exactly do you do in that case? How do you feel about it?   - Are you exposed to any triggers at work? |
| **Impact of the COVID-19 pandemic on self-management at work** | - Did the Corona pandemic change the way you deal with migraine at work? If yes, to what extent? |
| **Suggestions for improvement** | - What measures would help you cope (even) better with your migraine at work?   - What would the perfect workplace look like for you?   - How could your current workplace become a perfect one for you? |
| **Conclusion** | - Is there anything else you would like to add? |
