# Supplementary material for: Migraine self-management at work: a qualitative study
Source: J Occup Med Toxicol. 2024 Jun 4;19:22. doi: 10.1186/s12995-024-00421-w (PMC11149347; doi:10.1186/s12995-024-00421-w)
Supplement: Supplementary file 2 — Supplementary Material 2 [file 12995_2024_421_MOESM2_ESM.docx]

**Additional File 2 – Quotes**

| Quote no. | Quote |
| --- | --- |
| Quote 1 | “Whenever it starts at work, for example, and that’s really the case, my colleagues already see it. They notice how I look, how I walk, they see how I hold my head. And then, they always know: ‘Alright.’ And, well, we’re a great team, so I actually always get a lot of help, they do some work for me, or I can go home earlier.” |
| Quote 2 | “Well, I also have my supervisors, who simply support me. Just by leaving me some free space. (…) And I was the first one who could regularly work from home. That’s definitely a concession.” |
| Quote 3 | “So, I also notice that, even back then, this equalization [with a degree of disability] really helped me clarify, whenever I had stress at work due to sick days, I could just say: ‘Well guys, I have a disease, that’s a fact, and you can’t just ignore that now. It’s a fact and we all have to try to come to terms with it somehow.’” |
| Quote 4 | “Last year, when I was working for two months, the teachers’ council was actually very helpful, and I’ve also had a talk with them. (…) Well, at first I was a bit afraid of the conversation, because it’s like this: You have to get very personal. (…) I wanted to do it so that everyone could understand it. But afterwards, it felt so good to know that some colleagues know more about the situation and are very empathic. That has really encouraged me or simply helped me.” |
| Quote 5 | “Well, if it’s just internal telephone conferences where things discussed can be read in a protocol afterwards, you can absolutely excuse your absence and say: ‘No, this is stressing me out too much today, I’d prefer doing my day’s work in peace.’” |
| Quote 6 | “Sometimes, I just have to evaluate something on a piece of paper, and as soon as I can’t look at the screen anymore, I can read and cross things out on paper. That’s still possible or it works when the tablet is taking effect, rather than working at the screen. I can arrange this by myself.” |
| Quote 7 | “Concerning working from home, I’ve found that I just need to follow my rhythm. Then I can manage it and the chance that a migraine attack is triggered is much lower. You know, as I said before, I really start at 7 in the morning and do most of my work in the morning, because that’s when I can work best. (…) But also to say: ‘I wake up and I have a headache in the morning, so I’ll just stay in bed for another hour or two and start at 10.’ Simply having so much flexibility.” |
| Quote 8 | “For me, it’s easier to slow down a bit when I’m not feeling well at home. It’s easier than going to my colleague at two or half past two and say: ‘I’m going home now.’ Here [at home] I can just say: ‘Alright, I’m ill now, see you!’ and then I turn off my computer and I’m out. It’s different than leaving the office while everyone else is still working there.” |
| Quote 9 | “As I said, I was very sensitive to light, so when I was sitting alone in my secretariat, I took the liberty of lowering the blinds a bit so that I could simply open my eyes more easily and could work a bit because otherwise, I wouldn’t have been able to open my eyes at all.” |
| Quote 10 | “And, yes, I have a desk that is adjustable in height. It works quite well against tension in neck and shoulders. It doesn’t necessarily prevent migraines, but it helps, of course, since you’re always really tense anyway because of the pain in your head.” |
| Quote 11 | “I’ve also experienced and heard that there were explicit claims: ‘We’ve got back pain as well’ or ‘Sometimes, I’ve also got a headache and I don’t act like this’. If I had been absent for a day, it was often regarded as skipping work. So, I think there was little understanding of what migraine meant and to what extent it could lead to absenteeism.” |
| Quote 12 | “I mean, my colleagues are quite understanding, also my boss to some extent, but well, as soon as you’re there, you’re basically there. Nobody goes especially easy on you, you are challenged. So, nobody really takes it into consideration.” |
| Quote 13 | “There was no real understanding for this measure here, for the rehab, because I seem to be healthy obviously. It’s not a disease that you can necessarily see. Or also when I call in sick during work hours, my head of department sometimes says: ‘But you look quite normal.’” |
| Quote 14 | “But otherwise, nobody helps you. As I said, when you’re standing there alone and your colleague isn’t around. And the people [customers] who come there want to discuss their stuff, nobody takes you into consideration. It's not like people know how bad I'm feeling at that moment.” |
| Quote 15 | “There are usually four of us in the office. (…) And I don’t really think it needs to be so warm in there, because sitting in such a warm room doesn’t do my head any good either. I like to air the room sometimes and my colleague obviously starts to freeze then.“ |
| Quote 16 | “Well, I personally have a very hard time with screens, because they trigger my headaches, and I can’t escape this kind of work environment anymore. I mean, if I worked somewhere else, I’d be sitting in front of a screen just as much.” |
| Quote 17 | “What factors at work complicate dealing with migraine? – (…) If we are the host, we have to be available and approachable all day. (…) We have to arrive the evening before, greet the guests, look after them. And the next day, we have to be available all day during events, I think that’s extremely exhausting.” |
| Quote 18 | “Otherwise, customers can just come in without an appointment. That means I’m always interrupting my work, putting things aside and serving customers first, greeting or advising them, and much more. That’s definitely stress. Not the customers themselves, but having to keep interrupting work.” |
| Quote 19 | “So, a very big problem, for example, is that I also have to work on the weekend every five weeks. And that throws me off track, for example. That’s when I actually already know that I’m going to have a migraine the next day, because everything is messed up.” |
| Quote 20 | “I have to leave the realm of structured work every time or especially when I have to intensively analyze data all day and maybe also have time pressure to deliver results by the evening or when unforeseen events occur. I would say that these are situations that tend to favor it.” |
| Quote 21 | “Or sometimes, it [the migraine] develops so badly that I still finish my service, try to push it through. Because I also know that there is no substitute, nothing at all.” |
| Quote 22 | “My employment contract says that I only get my salary when I’m working. So when I’m sick, I don’t get paid, on vacation I don’t get paid, which is why I always go to work.” |
| Quote 23 | “Otherwise, I don’t think work triggered the migraine. Because for me, it has always happened over the years. Every few weeks, whether I’ve been on vacation or here at home, whether we’ve actually gone on a trip, whether I’ve gone to work. I hardly remember a vacation without at least one day of migraine where I [didn’t] lie in bed for one day. So that has always been the case.” |
| Quote 24 | “Corona is really bad, but for me personally, I always say that Corona is good for me. Simply because this ultimately leads to the decision that I can work completely from home now (…). And with that, I just have even more freedom in organizing my workday than before. That’s good for me.” |
| Quote 25 | “And for me, it’s always important that my life is somehow calm and orderly. And of course, it’s convenient for me that I can be at home, that there are relatively few colleagues in the office, that at most half of my colleagues are there. A lot of them work from home. And, yes, there’s no travelling. So overall, it has become quieter. And that’s actually good for me.” |
| Quote 26 | “And besides, I can also close the door for a longer time, we can also do that due to the pandemic now. Otherwise, we have this open-door-policy, that’s some kind of special case. It’s alright, but now I can also take the liberty of closing the door and then maybe feel like nobody sees you, I mean you’re not lazing around, but you can always do two or three stretching exercises.” |
| Quote 27 | “Right now, it’s a little different due to Corona, because it’s appointments only. I think that’s very convenient because I can prepare for each customer. They have to register with me beforehand, and then I can also plan it better according to my day. Then I know, okay now you can have a cup of tea in peace, take another breath before the next customer comes.” |
| Quote 28 | “Then I got the opportunity to work in a retirement home, and I wanted to start very slowly, but also due to the pandemic I noticed that it was more and more work for me. Also with the lockdown and the fact that it became more difficult for the residents to have contact from the outside or that the contact decreased, so I had more work to do. But that actually felt good because I just realized that I can do a lot. It was some kind of positive attitude towards life. So yeah, it has been good for me.” |
| Quote 29 | “In addition to the regular daily ward routine, which is already very stressful and short timed. You have many patients that you need to keep an eye on. And at some point somebody comes and says: ‘Yes, I’ve had contact with a corona patient or a person with corona outside the clinic’ and then the real stress starts for me, you know? Isolating, testing, that’s incredibly stressful, nothing is properly regulated here. And that’s something that throws me off track.” |
| Quote 30 | “Yeah, well, it [the migraine] got worse at times because I was also wearing the mask and I think I was just not getting enough oxygen, and also, you really have to speak louder and more accentuated. And that’s just exhausting. So, the first week, when I was wearing the mask all the time, I actually only had headaches and nausea.” |
| Quote 31 | “Because all the indicators got better. And then it was said that the office should be more populated again. So I was supposed to leave my, let’s say, comfort zone again. And even though I know that my team supports me, and I’ve talked to my boss about the fact that I’ll probably have to work from home more than three days in the future. That put a lot of mental strain on me and also triggered my migraines.” |
