# Supplementary material for: Migraine self-management at work: a qualitative study
Source: J Occup Med Toxicol. 2024 Jun 4;19:22. doi: 10.1186/s12995-024-00421-w (PMC11149347; doi:10.1186/s12995-024-00421-w)
Supplement: Supplementary file 3 — Supplementary Material 3 [file 12995_2024_421_MOESM3_ESM.docx]

**Additional File 3 – Migraine self-management strategies at work**

*What do participants do to manage their migraine at the workplace?*

Preventive strategies

Prevention aims at avoiding migraine attacks and to be prepared to swiftly respond to a possible upcoming attack. According to participants, the first aim seemed to be attained primarily by avoiding migraine triggers*,* which induced or intensified an attack. One frequently mentioned trigger was stress. The participants reported that the reduction of stress was feasible by integrating rest periods into their working day (e.g., by deliberately starting to work earlier), taking (digital) breaks, or restricting their availability (quote 1). Fixed daily structures or routines also seemed relevant to reduce stress and were achieved, e.g., through regular breaks and fixed mealtimes (quote 2). Some participants reported to have reduced their working hours or to have changed their job to reduce stress which triggered migraine (quote 3). Other triggers that participants reported to avoid were physical stimuli such as noise, odors, and bright light. For example, one teacher shared that she tried to avoid very noisy situations at school (quote 4).

To further create a workplace that was compatible with migraine and its management, communication with employers or colleagues seemed to serve as a preventive SM strategy. Through communication, agreements could be made, e.g., with respect to break times or remote working options. For example, one participant shared that his employer allowed him to have time for relaxation techniques during working hours (quote 5). Another participant told us that he negotiated work tasks with his employer to ensure that they exert no adverse effect on his migraine (quote 6).

To be able to quickly respond to an acute migraine attack participants carried an emergency kit with medication (especially triptans)) with them. Notably, even the awareness of being prepared for an attack was thought to help prevent attacks (quote 7).

Acute strategies

Acute strategies refer to strategies that are used to effectively respond to a migraine attack. A frequently mentioned strategy was taking medication. Adequate and rapid use of medication served to get through the working day despite migraine symptoms (quote 8) and to be able to attend important appointments (quote 9). Another strategy was taking sick leave – either not going to work at all or by leaving the workplace later in the day. In this respect, some participants emphasized the importance of full recovery from the attack to prevent a renewed attack (quote 10).

In addition to taking medication and withdrawing from work, calming down during the acute attack also emerged as a major coping strategy. This could consist of taking a short break, for example to use relaxation techniques such as progressive muscle relaxation or autogenic training (quote 11). Relaxation of the eyes (e.g., by not working at a computer screen or wearing sunglasses) was also reported to be relevant in this regard (quote 12). Relaxation often implied temporary withdrawal from the workplace. This short retreat took place, for example, in the break room. When a break room was lacking, withdrawal to the bathroom or to a quiet area was reported (quote 13). In contrast to relaxation, distraction in the acute migraine phase was also mentioned as being helpful. For example, one nurse shared that she was sometimes that distracted by working with patients that she did not notice the migraine attack anymore (quote 14).

Finally, just like in the context of preventive measures (see above), communication was perceived to serve as an acute SM strategy at work. Communication referred to seeking dialogue to gain understanding for the migraine from colleagues and supervisors or to ensure that one is not disturbed in the acute situation (quote 15). According to one participant, this dialogue was utilized, for example, to warn colleagues that one is not completely fit and efficient currently and to ensure thereby that colleagues can adjust (quote 16) and be available for support if necessary (see following section).

Quotes

| Quote no. | Quote |
| --- | --- |
| Quote 1 | “Things that help me? Well, I try to be unavailable after a certain time in the evening, for example. […] Like, turning off my phone then, or having specific times, also on weekends, when I don’t read any business e-mails. Some kind of digital breaks. Or I go out during lunch break and deliberately leave my phone at home so that I’m not available and really try to perceive the break as such.” |
| Quote 2 | “Well, I actually always take breaks, because they’re so important. Just five minutes to take a deep breath in between appointments ‘cause if you don’t take breaks, you quickly find yourself in this spiral as a person who suffers from migraine. Then, a migraine attack builds up in the course of the day and this can easily knock you out for several days if you’re not careful.” |
| Quote 3 | “I really try to avoid stressful situations as far as possible. I’ve quitted good positions several times and looked for new jobs because I couldn’t handle the stress and the pressure anymore and because I was ill all the time and basically spent my free time in bed.” |
| Quote 4 | “No sports classes [as teacher]. I don’t have that either, because I try to avoid anything that is more noisy than it already is in class, or situations with several stimuli at the same time, plus noise and maybe light and things like that. And I’ve also arranged that with the school so that I don’t have to do that, for example.” |
| Quote 5 | “Well, I tried to reduce the migraine with the help of medical or non-medical measures. And with non-medical, I mean, like, relaxation exercises. But then again, to talk with the employer on site, as well, and to say: ‘I need the possibility to do so.’ And they were like: ‘Alright, we can do this, you can use half an hour or twenty minutes of your work time for it and do it regularly.’” |
| Quote 6 | “I have specialized in a certain subject in the department that has no immediate connection to my regular activity. Now, it was said that I should go back and do more of the regular activity again whenever I’m doing well enough. But they also told me to be careful and watch out and that whenever the migraine is triggered too strongly again, I should just talk with the bosses about it and that we would find ways to limit the strain.” |
| Quote 7 | “That’s why I always carry my backpack with me, everyone else here finds it hilarious. My medication is in there. I never leave the house without them. (…) So, if I knew I didn’t have them with me, I think I’d get worked up about it and I’d also get a headache.” |
| Quote 8 | “It’s something typical that I get a migraine after three or four hours in the office and then I’m like: Well now you’re here, you have to pull through somehow. Because if I leave then, the day is considered sick and I wouldn’t be able to drive anyway. So I throw in triptan and let’s go. That’s a classic for me, actually.” |
| Quote 9 | “Whenever I still have important appointments during the day and I know that I MUST work well, I instantly take triptan as soon as I feel a slight headache.” |
| Quote 10 | “I usually have migraine for three days. The first day is usually the worst. The other two are also quite exhausting. Going to work is still very hard then. I’ve done that before as well, I went back to work after the first day, with medication. And then I had another attack right away. So, I notice you really have to cure it first.” |
| Quote 11 | “I always try to counter it somehow with a relaxation method or with stretching, because sometimes, it’s just a tension headache at first and it then ends up as a migraine attack. Sometimes I can soften this tension headache just as it’s developing by doing stretching exercises or by walking a bit, or with things like [muscle relaxation by] Jacobson, you know, just tensing everything up.” |
| Quote 12 | “I’ve also had days where a migraine was building up or decreasing and I just put sunglasses on in front of my computer. (…) It’s like either I go home right away, because I can’t work in front of my screen anymore or I put these damn dark sunglasses on and try to work for another hour or two.” |
| Quote 13 | “Well, I still remember the time during my apprenticeship and in the work phase afterwards. Every time I needed some rest, I would actually go to the bathroom for some time. But I was always paying attention not to be absent for too long, so that nobody would notice. And that’s when I would close my eyes and take a quiet breath.” |
| Quote 14 | “Well, at work when I’m sitting at the computer the whole time I would say I feel it [the migraine] more strongly, compared to when I’m working with patients. Then I don’t really notice it so much, because I’m distracted in a way. And suddenly I’m like: ‘Oh well, it’s gotten better now.’” |
| Quote 15 | “You’re not feeling well, and you say: ‘Look, today’s a really bad day.’ So you just make it clear to the others: ‘Please, just leave me out of it a little bit right now.’” |
| Quote 16 | “Sometimes, I come to the store in the morning despite my migraine, but then I realize that I’m not as productive as I would be if I didn’t have migraine. So, I always say: ‘Well guys, today you’ll have to make do with half of my brain, the other half’s dancing the samba right now.’” |
